# Hospital unit costs in Jordan: insights from a country facing competing health demands and striving for universal health coverage

**DOI:** 10.1186/s13561-022-00356-0

**Published:** 2022-02-05

**Authors:** Eman A. Hammad, Ibrahim Alabbadi, Fardos Taissir, Malek Hajjwi, Nathir M. Obeidat, Qais Alefan, Rimal Mousa

**Affiliations:** 1 Department of Biopharmarceutics and Clinical Pharmacy, School of Pharmacy, University of Jordan, Amman, 11942 Jordan; 2 Health Economy Directory, Jordan Ministry of Health, Amman, Jordan; 3 Department of Internal Medicine, Faculty of Medicine, University of Jordan, Amman, Jordan; 4 Faculty of Pharmacy, Jordan University of Science and Technology, Irbid, Jordan

**Keywords:** Unit costs, Jordan, Healthcare  Financing, Low- and middle-income countries, Hospital efficiency, Universal Health coverage

## Abstract

**Background:**

Public providers in Jordan are facing increasing health demands due to human crises. This study aimed to benchmark the unit costs of hospital services in public providers in Jordan to provide insights into the outlook for public health care costs.

**Methods:**

The unit costs of hospital services per admission, inpatient days, outpatient visits, emergency visits and surgical operations were estimated using the standard average costing method (top-down) for the fiscal year 2018–2019. The unit costs per inpatient day were estimated for nine specialities and staff in Jordanian dinars (exchange rate JOD 1 = USD 1.41).

**Results:**

The average unit cost per admission in Jordan was JOD 782.300 (USD 1101.80), the per inpatient day cost was JOD 236.600 (USD 333.20), the per bed day cost was JOD 172.900 (USD 244.90), the per outpatient visit cost was JOD 58.400 (USD 82.30), the per operation cost was JOD 449.600 (USD 633.20) and the per emergency room visit cost was JOD 31.800 (USD 44.80). The specialities of ICU/CCU and OB/GYN presented the highest unit costs per inpatient day across providers: JOD 377.800 (USD 532.90) and JOD 362.600 (USD 510.70), respectively. The average salaried unit cost of staff depended mainly on year of employment. Nonetheless, the unit costs varied depending on the service utilization, type of service and organizational outlet.

**Conclusions:**

Knowledge of how unit costs vary across public providers in Jordan is essential to outline cost control strategies and inform future research. Institutionalization of the cost information system and high-level governmental support are necessary to generate a routine practice of collecting and sharing cost information.

## Background

Jordan is located on the east bank of the Jordan River in the north of the Arabian Peninsula in western Asia and had a total population of around 9.5 million in 2015 [1, 2]. The gross domestic product (GDP) in 2017 was about 27.4 billion Jordanian dinars (JOD), equal to 38.65 billion United States dollars (USD), and the per capita health care expenditure was JOD 255, equal to USD 359.8 [1, 3]. The total health expenditure was estimated at JOD 2.25 billion in 2017 (USD 3.17 billion), accounting for 8.6% of the GDP [3, 4]. The public health expenditure as a percentage of the GDP is 5.5%. The total pharmaceutical expenditure accounts for 2.88% of the GDP and about 25.9% of the total health expenditure. These levels of expenditure are considerably high in comparison with those of countries in the surrounding region and with a similar economic status [3–5].

Universal health coverage has been a strategic goal of the Government of Jordan for over three decades [6]. It strives to provide financial protection against illness or injury and access to quality health care for all residents. Similar to most countries in the Eastern Mediterranean, the health care sector in Jordan is heavily subdivided into multiple providers, including public, private and international charitable providers [7]. The main health care provider is the public sector, consisting of three providers: the Ministry of Health (MOH), the Military Royal Medical Services (RMSs) and two university-affiliated hospitals (UHs) [3]. Jordan’s MOH provides health care services for approximately 60% of Jordanians, including public sector employees and their dependents. The health services of the MOH are provided through the civil insurance programme (CIP), which is funded mainly (77.5%) by the Government of Jordan, specifically the Ministry of Finance and Planning and the Ministry of Social Development, and sourced mostly from tax revenues [3]. Access to the public services of the MOH is extended to vulnerable Jordanians, and the following groups are exempt from user fees at MOH facilities: children aged under six, individuals classified as poor, individuals living in areas classified as “less fortunate” or remote, blood donors and families of which one member is an organ donor. Thus, substantial resources are allocated to achieving universal health coverage goals, namely health service access and financial protection [8].

The RMSs also offer health care services to 1.7 million people in the security and civil defence, as well as military veterans and their dependents, accounting for 27% of the population. Military personnel and veterans pay very low monthly subscription rates to health services (USD 5), with no or minimal co-payment when services are delivered (USD 1 per clinic visit). Contracted civil or public and uninsured Jordanians as well as non-Jordanians can also be treated at RMS facilities. There are two public university-affiliated hospitals at the University of Jordan and the Jordan University of Science and Technology (JU). The UHs’ insurance programme covers 2.5% of the population, including referrals for the MOH, employees of public universities and their dependents and employees of private or public firms with a contractual agreement. Private self-paying patients can also receive health services from the JU and pay out of pocket; they are mostly uninsured Jordanians or non-Jordanians [3, 4].

Public providers are interconnected through an unstructured referral system. For example, CIP patients with specialized or complex health needs can be treated at the RMSs and UHs and reimbursed based on the “fee for service”. Vulnerable non-Jordanians, including refugees, can be treated after referral too and are covered by charities or international donors, such as the United Nations High Commissioner for Refugees (UNHCR) and the United Nations Relief and Works Agency for Palestine Refugees. In addition, many individuals and their dependents are enrolled in more than one insurance programme. Hence, there is a significant overlap in health coverage and the exact number of insured and uninsured people remain uncertain [3].. Nevertheless, around 32% of the Jordanian population are uninsured according to the Jordanian national health account and concerns about financial hardship and treatment delays are growing.

Moreover, the health care system in Jordan is committed to responding to human crises in neighbouring countries [6]. The conflicts in Syria, Iraq, Libya and Yemen and the economic slowdown across the Gulf Cooperation Council have increased the economic constraints. In 2015, the total number of refugees from Iraq, Libya, Yemen and Syria in Jordan was estimated at 2.5 million. Syrians account for almost half of the non-Jordanians (1.4 million), and 53% of the refugees are reported as being under the age of 18 years [4]. The majority of registered Syrian refugees in Jordan live outside refugee camps and in Jordanian communities; the remaining 16% of refugees, an estimated 660,000, live in formal camps. The UNHCR and other humanitarian aid organizations, such as the Jordan Paramedic Society, Jordan Health Aid Society and Caritas, with support from the MoH, have been providing free health care for Syrian refugees living within camps. Syrians residing outside them, particularly with a UNHCR-issued asylum seeker certificate, can also access the highly subsidized services (80%) of public providers, similar to uninsured Jordanians [9, 10].

Nonetheless, all residents, even those who lack formal health insurance coverage, can benefit from subsidized health care services in Jordan. A study assessed the outpatient and inpatient charges from the largest MOH hospital and stated that the Government of Jordan covers almost 90 and 70% of direct medical costs for CIP-insured and uninsured Jordanians. In addition, Jordanians who lack formal public insurance coverage or the means to pay for health care may receive medical assistance through the Royal Court and other agencies. Vulnerable refugees and non-Jordanians receive health services that are supported fully by the UNHCR and other charitable organizations based on a vulnerability assessment framework [11]. It is worth noting that, in previous years (2011–2016), the MoH provided free health care for all Syrian refugees. This was a great burden on the health care system, but Jordan continued to seek ways to maintain access to health care and appealed for international aid. In response, a USAID-coordinated intervention established a multiple-donor account in 2019 with an estimated fund of USD 85 million, which supported Jordan in continuing to provide Syrian refugees with access to health services, with subsidized rates similar to those for uninsured Jordanians [10, 12].

Due to the availability of health services through several public and private programmes in Jordan individuals are able to access health services through public and/or private facilities and to have more than one type of insurance. The out-of-pocket expenditure representing direct payments from individuals to providers when receiving health services is rising, accounting for 27.8% of the total health expenditure in 2017 [3]. Private out-of-pocket expenditure represents 87.1% of the total out-of-pocket expenditure (JD 622 million) [3, 6]. A secondary analysis undertaken as part of the follow-up to the 2017–18 Jordan Population and Family Health Survey (JPFHS) implemented by the Department of Statistics (DOS) reported that most individuals without insurance seek care in private facilities, where they are much less likely to receive free treatment and more likely to pay higher costs, for both inpatient and outpatient treatments [13]. Hence, access to quality care and financial risk protection should be strengthened when seeking further steps towards universal health coverage. Therefore, assessing health costs locally and realistically and outlining costs across providers in the public sector are necessary tasks to facilitate accurate planning and budgeting as well as better financing for universal health coverage [4, 7, 14].

Estimates of unit costs are lacking, particularly for developing countries, due to data availability and the limited practice of evidence-based policy making [4, 15, 16]. Jordan, where economic findings are rarely a determinant of health policies or resource allocation, is no exception [17]. This study aimed to benchmark the average unit costs across multiple public providers in Jordan to provide useful insights into the outlook for public health care costs in Jordan and identify ways to control costs and move towards better access to quality health care.

## Methods

The cost analysis of health care from the perspective of public providers in Jordan was performed using a top-down costing method. Unit costs were defined as the cost of one unit of “output”, including admission, inpatient days, outpatient visits, emergency visits and surgical operations [16, 18–20]. The unit costs per hospital output were estimated using standard average costing following a top-down approach [19–22]. The total health expenditures of a provider were divided by the total output produced to determine the unit costs [19–23].

The formula used was the following:

Average cost = total expenditure ÷ total output units. Total expenditure =  ∑  *C*_*drugs*_ +  *C*_*salaries*_ +  *C*_*medical and non* − *medical  supplies *_ +  *C*_*food and housekeeping*_ +  *C*_*resource developement and training*_ +  *C*_*medical and non* − *medical equipment*_ +  *C*_*administration*_ +  *C*_*utlilities and overhead  expenses.*_

The total health expenditures were defined as the value of the health goods used for the production of all the activities of a provider [14]. They were allocated based on the proportion of resources consumed from the budget of a provider in the delivery and production of services within inpatient, outpatient and emergency departments and surgical units [19–22]. The data were collected and analysed between September 2015 and January 2018.

### Data sources and collection

Data were extracted from multiple sources to enhance the study’s validity and reliability. Published as well as unpublished official reports and providers’ financial records were reviewed. Reports were obtained from the Health Economic Directorate, public affairs offices, the Ministry of Finance Budget, the planning and health insurance divisions and the finance/IT departments of each provider (MoH, RMSs and UHs). Data were also extracted from the National Health Account Technical Report published in 2019 (the most recent issue) and the annual statistical reports for 2016–17 from each of the providers. Data were not available for the 48 individual hospitals but at the level of the three sets of providers, including the MoH health economic directorate (relating to all 32 hospitals) and the RMS public affairs office (relating to all 14 hospitals). Hospitals under these providers are managed centrally in terms of operation, cash flow and management. Data were also collected directly from the two UHs: the Jordan University Hospital and King Abdullah University Hospital. These are public institutions but operate with increased autonomy, authority and independence to manage their operations [3, 4]. Data were obtained from the chief financial managers of the three sets of providers (MoH, RMSs and the two UHs) and verified by the Vice Director for Administrative Affairs. Table 1 summarizes the data collection and sources.

**Table 1:** Data sources ^b^

Data collected	Source(s)
Total expenditures per provider (inpatient & outpatient)	Providers financial records, NHA, the Health Economic Directorate, public affairs offices, the Ministry of Finance Budget, the planning and health insurance divisions, and the finance departments in each provider
Total expenditures per speciality	Providers financial records, Providers annual statistical reports
Output units per provider (no. admissions, visits to outpatient, emergency room, no of operations)	Providers annual statistical reports, NHA
No. inpatient days per speciality	The finance/IT departments in each provider, The MOH the Health Economic Directorate, and the public affairs offices in each provider
Hospital bed	Providers annual statistical reports, NHA
Average length of hospital stay	Providers annual statistical reports, NHA
Staff salaries^a^	The finance/IT departments in each provider, Health Economic Directorate& planning and health insurance divisions, (MOH), public affairs offices (UH, RMS), Jordan civil service bureau, Health Professional association (Medical, Nursing, pharmacist).

^a^ Unit costs of staff present basic salary, benefits, gratuities, and social charges. Information about the staff salary ranked system of each provider was obtained from their chief financial managers and human resource departments. The estimated ranges of unit costs of staff were face validated after discussions with conveniently selected staff (two or three staff members in each position, rank or band) from each provider. ^b^ Data were available for the three sets of providers from the MoH health economic directorate (relating to all 32 hospitals) and the RMS public affairs office (relating to all 14 hospitals). Data were collected at individual hospital level for both UHs

### Unit costs of hospital services

The unit costs were calculated using the average cost method relevant to hospital-based services in the three types of public providers. The total expenditure of a provider was divided by the output units [22, 24, 25]. The unit costs per inpatient day included the day of admission but excluded the day of discharge or death. The total inpatient days were calculated by adding the daily patient census days relative to 365 days in all the departments of the study providers. An inpatient day was defined as a day during which a patient is confined to bed and stays overnight in the hospital. The total number of bed days was obtained by multiplying the total number of beds by 365, presenting the maximum number of inpatient days if every available bed was occupied every single day, that is, the maximum number of inpatient service days that can be offered by the providers annually. The cost per bed day represented the averaged costs of a hospital bed, whether occupied or not [22, 24, 25], whilst the cost per inpatient referred to the average cost of actual inpatient days per provider, that is, when a patient is admitted, accounting for the occupancy rate/average length of a hospital stay [26]. The cost per surgical operation did not include pre- or post-operative costs.

The unit costs per speciality were estimated in relation to nine specialities: orthopaedic, general and specialized surgery, internal medicine, paediatrics, ENT, neonatal care, ophthalmology, OB/GYN and ICU/CCU. These received the largest numbers of patients across providers. Specialities that require similar labour and capital inputs to treat patients with similar maladies were grouped together [19, 21, 26].

The total expenditures were allocated to specialities based on the proportion of inpatient admissions. The unit cost per inpatient day per speciality was obtained using the following formula: cost per inpatient day_I_ = total expenditure _inpatient_ * [inpatient admission_I_ / total inpatient admission] ÷ inpatient days_i_ [19, 21, 26]. Inpatient days_i_ is the number of inpatient days for a medical speciality. The average unit costs of the three sets of providers were pooled, dividing the total expenditures of the three sets of providers for producing a service, over the total number of services produced across the three sets of providers [22, 24, 25].

The unit costs were estimated in JOD. The exchange rate was USD 1.41 based on the quotation from the Central Bank of Jordan on 24 October 2020. The health expenditures were inflated to 2019; the inflation rate was averaged as 5.1% from 2013 to 2020, as reported by the Hashemite Kingdom of Jordan Department of Statistics 2020.

### Unit costs of medical and ancillary staff

The unit costs of staff represented the average salary range per hour. This included the basic salary, benefits, gratuities and social charges. The number of working hours for each profession, subtracting annual, statutory and sick leave days from week days per annum, were estimated at 260 days. The unit cost per hour was estimated by dividing the average salaries by working hours [27]. Information about the staff salary ranked system of each provider was obtained from their chief financial managers and human resource departments. The estimated ranges of unit costs of staff were face validated after discussions with conveniently selected staff (two or three staff members in each position, rank or band) from each provider.

## Results

Background information about the study providers is presented in Table 2. The total number of hospitals included in the study analysis was 48, with a total of 9235 beds and 26,753 salaried health staff, including physicians, dentists, pharmacists, nursing staff and midwives. The total numbers of admissions and outpatient visits were 659,424 and 8,188,631, respectively. The average occupancy rate was 71.3%, and the average length of hospital stay was 3.7 days. In terms of the numbers of hospitals, beds, admissions, outpatients, emergency room visits, inpatient days, workforce and expenditures, the MOH is the main provider of health services. Notably, the RMSs demonstrated the highest death rate, average length of hospital stay and number of surgical operations [19, 23, 26].

**Table 2:** Background information on public health providers in Jordan

	MOH	RMS	UHs
Hospitals	32	14	2
Beds	5177	2917	1141
Admissions	374,818	190,364	91,242
Death rate	1.8%	3.3%	1.4%
Out-patient visits	2,978,989	442,911	772,970
Occupancy rate	65.8%	78.6%	72.8%
Average length of stay (days)	3.2	4.3	3.7
Surgical operations	88,305	102,489	41,339
Emergency room visits	2,933,928	1,081,635	193,110
Inpatient days	1,187,236	796,861	293,065
Healthcare Personnel^a^	16,464	9491	2374
Contribution to health expenditures	62.6%	27.2%	9.9%

*MOH* Ministry of Health, *RMS* Royal Medical Services, *UJH* University of Jordan hospital

^a^Includes physicians, dentists, pharmacists, nurses and midwives. Source: Annual statistical reports in each provider 2016–2017 and Jordan National Health Accounts 2019, Technical Report No. 8

### Unit costs of hospital services

Table 3 presents the unit costs by provider. The unit costs of the RMS hospital services were the highest among the providers, except for emergency visits. The UHs had the highest costs per emergency visit. On the other hand, the unit costs of the MOH were modest across all the estimates. It can be seen in Table 3 that, in Jordan, the average unit cost per admission was JOD 782.300 (USD 1101.80), per inpatient day was JOD 236.600 (USD 333.20), per bed day was JOD 172.900 (USD 244.90), per outpatient visit was JOD 58.400 (USD 82.30), per operation was JOD 449.600 (USD 633.20) and per emergency room visit was JOD 31.800 (USD 44.80).

**Table 3:** Unit costs of hospital services per provider in Jordan

Unit costs (JDs)	MOH	RMS	UHs	Average^**a**^
Cost per admission	557	914	740	782.3
Cost per inpatient day	175	277	230	236.6
Cost per bed day	119	207	162	172.9
Cost per outpatient visit	24	71	62	58.4
Cost per operation	402	548	399	449.6
Cost per emergency room visit	22	26	47	31.8

^a^The average unit costs of the three sets of providers were pooled, dividing the total expenditures of the three sets of providers for producing a service over the total number of services produced across the three sets of providers

Table 4 estimates the unit costs of inpatient days per speciality in JOD. ICU/CCU and OB/GYN presented the highest unit costs per inpatient day across providers, respectively. These were followed by the unit costs of ophthalmology, estimated at USD 496.62 and neonatal care estimated at USD 438.31. Orthopaedics demonstrated the lowest unit costs for inpatient days across all the providers.

**Table 4:** Unit costs of inpatient hospital days per specialty

	Cost per inpatient day (JODs)			
Speciality	MOH	RMS	UHs	Average
Orthopaedic	169.8	161.1	168.1	163.7
General and specialized surgery	282.3	217.5	215.4	242.7
Internal Medicine	238.4	265.1	245.1	253.8
Paediatrics	240.8	280.1	286.4	265.9
ENT	220.3	392.1	359.5	328.4
Neonatal care	365.1	417.8	235.1	342.2
Ophthalmology	322.2	340.0	384.2	352.6
OB/GYN	320.3	419.1	342.1	362.6
ICU/CCU	327.8	413.9	398.7	377.8

*ENT* Ear, Nose and Throat, *ICU/CCU* Intensive Care Unit/Cardiac Care Unit, *OB/GYN* Obstetrics and Gynaecology, *MOH* Ministry of Health, *RMS* Royal Medical Services, *UJH* University of Jordan hospital

### Unit costs of medical and ancillary staff

Table 5 presents the average salary for each rank of staff per hour. With every year of employment, the paid wages increase by an estimated 10–20%. This is accompanied by moving up a band or structured rank. The highest appointment level is often granted after 15–20 years of full-time employment. For the MOH, moving up bands/grades is mainly based on years of experience. The RMSs follow in-house residency and training programmes involving profession development and research publications. In addition to the years of employment, teaching hospitals’ wages are based mainly on teaching and academic roles. Roles involving direct patient care services are reimbursed by providers independently in relation to the number of patients or the number of procedures and consultations performed.

**Table 5:** Unit costs of medical and ancillary staff

Profession	Range JODs per hour
**Medical staff**	
**MDs (Doctor of medicine)**	
Postgraduate registration year	1.75–2.5
MD Intern (1–4 years) post registration year	7.5–20
Fifth year MD resident	10–22
Consultant^a^	13–57.5
**Nurses**	
Assistant nurse	4.05–11.9
Registered nurse (first degree)	6.68–37.5
**Ancillary staff**	
Pharmacy technician	4.38–12
Registered pharmacist	7.5–26
Administration, finance departments	4.9–22.5
Laboratory, Radiology, Anaesthesia Technicians	5.4–14.75

*MD* Doctor of medicine

^a^For teaching and professional roles

## Discussion

This is the first study to estimate the unit costs of hospital services from multiple public providers in the region. Previous studies conducted in Jordan and nearby countries have assessed health costs only on the level of a single facility or disease rather than on the health system level [19, 23, 26]. The unit costs of hospital services varied widely depending on the service utilization, type of service and organizational set-up. This is in agreement with studies from middle- to low-income countries [16, 20, 22, 23, 28]. However, the average estimates of unit costs in this study are higher than the previous local estimates obtained from a single MOH hospital in Jordan in 2017 [26]. For instance, adjusted to the same fiscal year (2019), the cost per admission amounted to almost USD 1100 compared with USD 715, whilst the cost per inpatient day was USD 334 compared with USD 159. This is potentially in line with the WHO, which has highlighted the increase in health costs as a key challenge to achieving better access to health care services in the Eastern Mediterranean region [7].

The unit costs of hospital services in the military services (Table 3) were the highest among the providers. The RMS hospitals are predominantly tertiary providers and are oriented mainly towards specialized inpatient care, such as surgical referrals, particularly for head, cardiovascular and paediatric surgery [6]. In addition, the numbers of specialized medical consultants and high-skilled health workers are estimated to be higher for RMSs: 3677 compared with 1380 in MOHs. Additionally, the RMSs operate a number of specialized hospitals for paediatric services, heart surgery and kidney diseases. However, the cost per outpatient attendance was found to be the highest for RMSs, with their service utilization being relatively the lowest (Table 2). Thus, the cost drivers are not clear and are potentially related to the labour costs, prescribing patterns or capital costs [6, 26]. Investigating the cost drivers on the level of the provider is essential to ascertain the reasons for cost variances or the areas requiring efficiency improvements [15, 24]. Future research is warranted.

On the other hand, the lowest cost per operation was found in teaching settings. In total, UHs performed over 41 thousand surgical operations (Table 2). The lower costs could be due to the increased service output and lower labour costs in teaching hospitals than in tertiary settings, that is, more operations performed by residents or fellows. This has been outlined in similar studies [16, 20, 22, 23]. Thus, the type of provider is potentially an influential factor in hospital costs.

Also, it is notable that the cost per emergency room visit, was the highest in UHs. UHs are heavily focused on outpatient services that are not available at weekends, during holidays and after working hours. The higher costs might reflect the increased health resource utilization in acute and emergency settings outside normal working hours [4]. Therefore, telehealth and the diversion of patients to walk-in clinics or contracting primary care with MOH 24-h primary care centres might present potential cost-saving strategies [26, 29].

The unit costs varied across specialities (Table 4). Those with higher capital investment, complex care modalities and lower outputs presented the highest costs, specifically ICU/CCU, OB/GYN, ophthalmology and neonatal care. Previous research in Jordan has highlighted that fixed costs, including human resources and capital costs, are the largest components of hospitals’ total operation costs [26]. This is in agreement with previous studies conducted in other countries [15, 21, 26, 27]. Additionally, managers in specialities with the lowest number of beds or with an occupancy rate less than 15% were advised to consider technical efficiency by shifting patients to day care services or adopting an on-call-based staff system. These approaches have the potential to enable cost saving in costly care centres. However, future research is recommended to gain a better understanding of how these strategies might be influenced by patient- or disease-related cost drivers. The development and implementation of treatment and prescription guidelines can also help to control cost variability and service quality across providers [3, 6, 28].

This study is the first to benchmark cost ranges of medical and ancillary staff (Table 5). These estimates can be useful for future costing studies and to inform efficient service delivery methods. Labour costs can be controlled by directing the mid-level workforce to conduct outreach activities, data collection, paperwork or patient follow-up.

Our study sheds lights on the variability of the cost of treating patients across the public health system of Jordan. Identifying the differences is critical to inform reimbursement and strategies for universal health financing and social protection of Jordanians and refugees [7, 23]. The investment needed to strengthen health systems in middle-income countries might be substantial considering the socioeconomic constraints in place. However, without systematic and realistic assessment of the costs, managers cannot plan strategically for both better access and better health quality. In addition, donors potentially misestimate the funds necessary to attain these goals. Therefore, the improvement of efficiency and the adaptation of cost information system will be crucial to achieve health access and financial protection goals for refugees or hosting communities in Jordan [30]. Effective partnerships and coordination among public providers are demanded. Improved linkage to primary care and preventive treatment programmes and strengthened capacity for emergency and triage services are essential for optimizing referral mechanisms and service contracts.

### Challenges for cost information research

It is important to outline the contextual challenges that were faced during the course of the study. The leading challenge concerned the willingness to share cost information, quality of recording systems and availability of data. Managers were resistant to sharing information for research purposes. They perceived this as uncommon practice with unpredictable consequences. Obtaining approval to access data took 18–24 months due to several discussion meetings and background as well as security checks. The study team was not granted direct full access to hospitals’/providers’ records or data system, and all the data were obtained through meetings with the chief financial manager of each provider. Thus, collating and acquiring data were time consuming. Data were extracted from various sources and were often not readily available on the provider level. However, these challenges are similar to those reported in other developing countries [16, 20]. Hence, there are a number of lessons to share. The institutionalization of cost information is necessary in developing countries [15, 16, 19, 23, 31]. Health care providers should be obliged to adopt a robust cost information system to enable cost analysis and value-based budgeting. In Jordan, the General Secretariat of the Higher Health Council institutionalized the National Health Account in 2007. This will potentially support the collection and production of robust data and information [3, 6]. Political support for generating laws to legitimise the role of cost information in budgeting and reimbursement decisions is required too. This would enhance acceptance and information-sharing practice. Additionally, local and international multidisciplinary discussions, peer published evidence and training for local managers can help to prompt cost information sharing, transparency and experience exchanges [32, 33].

### Limitations

The unit cost calculations in this study were based on the average top-down method rather than micro costing on the patient or the disease level. This approach carries the risk of underestimating the units with high capital investments, fewer patients and high maintenance costs [22, 23, 34]. Additionally, data were not collected for each of the 48 hospitals individually due to the data availability and the organizational structure of the providers. The average unit costs presented here are per provider rather than per hospital. Thus, the variability of costs within a provider type or on the facility level remains unclear. Future studies using mixed methodologies including micro-costing, expert elicitation and qualitative interviews will enable further understanding of cost drivers. Additionally, the estimates of staff unit costs benchmarked the range of salaried costs only. There are other costs related to training, specialty, year of practice, incentives and experience. Thus, the cost drivers related to the staff unit costs across types of providers, departments and professions cannot be addressed fully. Future studies are recommended too.

The previous research on hospital unit costs from Jordan presented detailed estimates relevant to cost types and categories, such as labour, overheads, medications, supplies and so on. This potentially enabled more precise estimates for cost drivers and the distribution of various cost categories to the overall hospital budget and care centres. These details were readily available and collected for in-house auditing purposes for one hospital [26]. In Jordan, costing information is not recorded or utilized routinely. A routine health service costing system on the provider and facility levels should be promoted through expert support networks, developing evidence-based cost catalogues and involving stakeholders in determining the scope and use of cost information in evidence-based decision making [16, 31].

This study presented cost estimates for a selected number of specialities. The specialities with the most patients across providers were recognized after discussions with the chief financial manager of each provider. The cost drivers in smaller or specialized services remain unknown. For instance, all psychiatric beds are located in four mental hospitals, two of which are operated by the MOH, one is run by an RMS and one is a private hospital. There are no psychiatric wards or beds in university facilities. Hence, in this study, the cost of psychiatric care was not presented. Future studies assessing the costs of other specialities, diseases or patient sub-group are warranted.

## Conclusions

It is important for health policy makers and managers to produce local and realistic cost estimates when delivering health services to ensure sustainability, quality and accurate budgeting. Knowledge of how unit costs vary across public providers in Jordan is essential to outline cost control strategies and inform future research. It is also crucial to plan realistically the resources needed for universal health coverage. The institutionalization of a cost information system and high-level governmental support are needed to generate a routine practice of collecting and sharing cost information.

## Data Availability

The datasets used and/or analysed during the current study are available from the corresponding author on reasonable request.
